# Morphogenesis-coupled DNA repair - in mammalian embryogenesis, morphogenesis and DNA double strand break (DSB) repair are carried out simultaneously to ensure normal development

**DOI:** 10.1093/jrr/rrae028

**Published:** 2024-05-07

**Authors:** Asao Noda

**Affiliations:** Department of Molecular Biosciences, Radiation Effects Research Foundation, 5-2 Hijiyama-Park, Minami-Ku, Hiroshima 732-0815, Japan

**Keywords:** morphogenesis, DSB repair, CHD7, PTIP, COBRA1, BRIT1/MCPH1

## INTRODUCTION

Embryonic development in higher organisms is an irreversible process controlled by a strict and predetermined genetic program. In their early embryo, dramatic changes called ‘embryo morphogenesis,’ which involve cell division and migration of cell populations, as well as three-dimensional tissue organization, occur precisely within a fixed time slot. This elaborate mechanism has fascinated developmental biologists for many years. However, excessive stress from the external environment can disrupt this process. For example, certain toxic chemicals and radiation can inhibit development or cause abnormal morphogenesis. During evolution, organisms have developed molecular mechanisms to deal with problems in all phases of cell metabolism. Thus, mechanisms must be present to deal with genomic damage during embryonic development, while maintaining a predetermined timetable. To date, damaged cells occurring during embryonic development were only considered to be eliminated by p53-dependent apoptosis to prevent the development of malformations. In this short review, I summarize recent findings on the mechanisms of double-strand break (DSB) repair (or DSB stress response) coupled with morphogenesis using regulatory proteins like chromodomain helicase DNA binding protein 7 (CHD7), Pax2 transactivation domain interacting protein (PTIP), co-factor of BRCA1 (COBRA1) and BRCT-repeat inhibitor of TERT expression (BRIT1) as examples.

### Example 1: Chromodomain helicase DNA binding protein 7(CHD7)

**Fig. 1:**
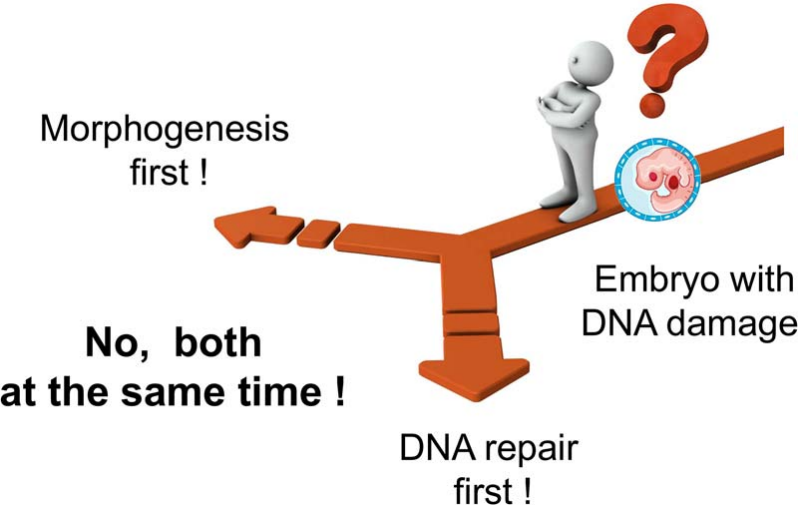
Decision of embryo with DSB damage.

Excessive radiation exposure can cause unrepaired DSBs in cell nuclei. These can be easily detected and quantified in apoptosis-resistant mesenchymal and stromal cells, such as fibroblasts [1]. In the process of screening for genes characteristic in cells bearing unrepaired DSBs, we discovered that *CHD7* transcription was upregulated in these cells [2]. We also confirmed ATM-dependent phosphorylation of CHD7 protein and its accumulation in DSB foci protein complexes after radiation exposure. CHD7 is a transcription factor ubiquitously expressed in adult tissues; it is also known to control neural crest differentiation and morphogenesis during early development [3]. Neural crest cells are a migratory cell population transiently located in the border region between the neural plate and epidermis during early embryogenesis. They form neural, skeletal, epithelial and mesenchymal tissues and participate in several organogenesis processes, such as the development of craniofacial architecture, brain, and heart. The neural crest is thus called the ‘fourth germinal lobe’ [4]. CHD7 is expressed upstream of the transcription factors Slug, Twist, and Sox9 in the mesenchymal-epithelial transition of the neural crest and is essential for the formation of migratory neural crest cells [3]. It is also expressed upstream of semaphorin 3A (SEMA3A) and ephrin receptors and regulates the guidance of neuroaxonal elongation in 9.5- to 11.5-day mouse embryos [5]. *CHD7* heterozygotes arising from *de novo* germline mutations show multiple developmental abnormalities in neurosensory organs, such as the eyes, mouth, nose, ears and brain, as well as heart malformations, indicating that gene haploinsufficiency induces fetal malformations. *CHD7* is the major gene responsible for the Coloboma, Heart defect, Atresia choanae, Retarded growth and development, Genital abnormality, and Ear abnormality (CHARGE) congenital malformation syndrome in humans [6, 7]. *SEMA3A* mutations are found in Kallmann syndrome, which resembles CHARGE [5]. Further, CHD7 is essential for neural crest induction from undifferentiated tissues. The fact that CHD7 localizes to the DSB site [8] and is phosphorylated in an ATM-dependent manner by switching its localization from promoter/enhancer binding to DSB binding [2] indicates that this transcription factor has both morphogenetic and DSB repair functions in the developing neural crest. The embryonic morphogenetic stage is highly sensitive to radiation, and excessive radiation induces malformations [9]. In mouse fetuses, induction of malformations peaks in 7- to 13-day embryos irradiated at 2 Gy. Although CHD7 is the most active during this period and drives morphogenesis, it may also drive concurrent repair in response to genomic stress. This coupling, or switching, between morphogenesis and DSB repair is attributed to ATM-dependent phosphorylation and the transient translocation of proteins to DSB sites. In case of excessive DNA damage or many cells bearing unrepairable damage, CHD7 remains at the DSB site for too long, thereby resulting in a reduction of its morphogenetic activity. Otherwise, cells with unrepaired DSBs could progress to mitotic arrest, preventing development. This is a plausible cause of radiation-induced fetal malformations and a good example of a morphogenetic transcription factor with DSB repair (response) function (Fig. 1).

**Table 1:** Protein factors involved in morphogenesis-coupled DSB repair

Gene product	Protein characteristics	Target tissue in morphogenesis	Distinctive domains	Associated diseases
CHD7	Promoter/enhancer binding	Neural crest cells	Helicase	CHARGE syndrome
PTIP	Pax2 binding	Intermediate mesoderm	6XBRCT	Renal coloboma
COBRA1	BRCA1 binding	Mammary epithelium	NR binding motifs	Breast cancer
BRIT1/MCPH1	Promoter binding	Neuroprogenitor cells	3XBRCT	Microcephaly

NR: nuclear receptor; CHARGE: coloboma, heart defects, atresia choanae, growth retardation, genital abnormalities, and ear abnormalities

### Example 2: Pax2 transactivation domain interacting protein (PTIP)

Paired Box Gene 2 (*Pax2*) is a transcription factor that controls intermediate mesodermal differentiation. *Pax2* KO mice are born without kidneys. They also lack the ureter, reproductive tract, oviduct, uterus, and vagina [10, 11]. Moreover, Pax2 controls eye and inner ear formation—in KO neonates, the eye cup cannot be closed, the optic nerve cannot be semi-crossed and becomes fully crossed, and inner ear hearing is defective. Therefore, *Pax2* is a causative gene for congenital malformations [12, 13]. PTIP was discovered as a Pax2-binding protein and was thought to bind the transactivation domain of Pax2 to promote transcription [14]. During the initial stage of kidney primordium formation from the intermediate mesoderm, Pax2 remains activated in the absence of PTIP. However, in the late stages of kidney formation, Pax2-activated gene expression is suppressed in the absence of PTIP. *PTIP* KO causes embryonic lethality in 9.5-day embryos [15] and arrhythmia and glomerular disease in conditional KO adults [16, 17]. PTIP is an adaptor protein carrying six BRCA1 C-terminal (BRCT) domains and is a protein that is typically associated with DSB foci [14, 18]. During DSB repair, PTIP binds to 53BP1 on DSBs, transiently suppresses end-resection, and promotes nonhomologous end-joining [19]. This complex of PTIP and Pax2 is required in kidney development [20].

### Example 3: Cofactor of BRCA1 (COBRA1)

COBRA1 is a BRCA1-binding protein [21] that promotes mammary gland development in a hormone-dependent manner [22]. Deletion of *COBRA1* suppresses mammary gland development. However, when *BRCA1* is also depleted (double KO), mammary gland development resumes (double negative shows somewhat positive), suggesting that the BRCA1/COBRA1 complex normally suppresses mammary gland development at the transcriptional level, timing its hormone-dependent activation [23]. As neither the RING nor BRCT domains are involved in the BRCA1-induced repression of COBRA1 function, the homologous recombination repair (HRR) function of BRCA1 and mammary gland development do not seem to be directly related. However, breast cancer may occur if the suppressive function of the BRCA1/COBRA1 complex is attenuated and abnormal mammary cell proliferation (hyperplasia) occurs owing to over-mobilization of BRCA1 to DSBs for HRR. In other words, this is another good example of morphogenesis coupled with DSB repair, considering that it normally regulates mammary gland development, while DSB repair occurs simultaneously in the event of damage.

### Example 4: BRCT-repeat inhibitor of TERT expression/Microcephalin (BRIT1/MCPH1)

The transcription factor BRIT1 was initially identified as a negative regulator of hTERT gene expression during somatic cell differentiation [24]. BRIT1 controls the transcription of target genes by binding to TRF2 and E2F. BRIT1 was also found to be a typical DSB foci factor shortly after its discovery because *BRIT1* encodes three BRCT domain repeats. Surprisingly, this gene is identical to *MCPH1*, the causative gene for microcephaly [25], indicating that it binds to telomere ends and radiation-induced DSBs, as well as regulates brain development [26]. BRIT1/MCPH1 regulates brain development by controlling the division of neuroprogenitor cells and temporal–spatial processes via mitotic checkpoint regulation. Thus, this key transcription factor regulating brain development and cell division also acts as a DSB stress response factor during radiation exposure.

## CONCLUSION

The above examples indicate that some of the key transcription factors controlling embryo development and differentiation are also involved in the DSB stress response or directly in DSB repair. They ensure normal development by simultaneously performing morphogenesis and DSB repair in the irreversible process of morphogenesis. Under excessive genome damage, the DSB response may reduce the driving force of morphogenesis owing to the mobilization of more proteins to the DSB sites, resulting in the induction of fetal malformations. The morphogenetic period of early development is a continuous biological process with a point of no return, in which the most complex life phenomena must occur precisely within a predetermined timeframe. To ensure this process, it is reasonable to assume that the key transcription factors have themselves acquired a DSB repair (DSB stress response) function during evolution. From this perspective, the p53 family of genes might broadly fall into the same category as p53, which is a stress-responsive transcription factor and malformation suppressor [27, 28]. However, p53 is a negative regulator that removes damaged cells during embryogenesis, whereas the morphogenic factors described above are positive regulators that promote the recovery of damaged cells. Furthermore, it has been suggested that some of these regulatory factors may also play a role in carcinogenesis [13, 29], as cancer could potentially result from a disruption in morphogenetic activity.

During evolution, organisms have successfully dealt with genomic damage during the most critical phases of life to maintain their continuity. This has led to the creation of proofreading mechanisms in DNA replication and the development of a transcription-coupled repair mechanism. Similarly, a mechanism must have been established for simultaneous morphogenesis and repair of genomic damage occurring in cells during early embryogenesis because of exposure to radiation or other stresses. Additional morphogenic factors with such DSB-stress response functions are expected to be identified in future.
